# Prolonged exposure to teixobactin generates cross-tolerance to other cell wall-targeting antimicrobials in *Enterococcus faecalis*

**DOI:** 10.1128/aac.00629-25

**Published:** 2025-11-18

**Authors:** Rachel L. Darnell, Melanie K. Knottenbelt, Olivia E. Rose, Caitlin S. B. Cleary, Francesca O. Todd Rose, Hannah Hodgkinson, Yuezhou Wu, Maytham Hussein, Tony Velkov, Susanne Gebhard, Gregory M. Cook

**Affiliations:** 1 Department of Microbiology and Immunology, University of Otago 626306, Dunedin, New Zealand; 2 Maurice Wilkins Centre for Molecular Biodiscovery, University of Auckland 1415, Auckland, New Zealand; 3 Milner Centre for Evolution, Department of Life Sciences, University of Bath 14289, Bath, United Kingdom; 4 Department of Pharmacology, Monash Biodiscovery Institute, Monash University 2541, Melbourne, Australia; 5 Institut für Molekulare Physiologie, Johannes Gutenberg-Universität Mainz 124421 , Mainz, Germany; The Peter Doherty Institute for Infection and Immunity, Melbourne, Victoria, Australia

**Keywords:** mevalonate pathway, cell envelope stress response, LiaFSR, cross-tolerance, *Enterococcus*, cell wall targeting, resistance, antibiotic tolerance, enterococcal polysaccharide antigen

## Abstract

Antimicrobial tolerance, i.e., the ability to survive despite growth inhibition, is an important precursor to the development of antimicrobial resistance. However, very little is known about the evolution of drug-induced antimicrobial tolerance. Teixobactin (TXB) is an antimicrobial peptide that targets the cell envelope precursors lipid II and lipid III in Gram-positive bacteria. We have previously shown that *Enterococcus faecalis* displays high intrinsic tolerance to killing by TXB, and this may aid in the development of TXB resistance in this species. Here, we demonstrate that prolonged exposure to TXB led to the emergence of enhanced tolerance, but not TXB resistance. Whole-genome sequencing of these TXB-tolerant mutants identified mutations in the mevalonate and Epa (enterococcal polysaccharide antigen) biosynthesis pathways, *hprK*, a key regulator of carbon metabolism, and *liaF*, a negative regulator of the LiaSR cell envelope stress response. Increased susceptibility to TXB in single gene deletion mutants of the sensor kinase *liaS* and cognate response regulator *liaR* provides further support for a novel role of the LiaFSR cell envelope stress response system in TXB tolerance. Finally, we demonstrate that constitutive hyper-signaling of the CroRS and LiaSR cell envelope stress responses is consistent with broad tolerance to TXB and the clinically relevant antimicrobials daptomycin and ampicillin, suggesting expression profiling of the cell envelope stress response may serve as a key indicator of antimicrobial tolerance in *E. faecalis*.

## INTRODUCTION

Antimicrobial tolerance (AMT) is the ability of a microorganism to survive but not proliferate during high-dose antimicrobial treatment (1). Mechanisms of AMT have been linked to many different cellular processes and can be intrinsic or acquired at the individual or population level (2, 3). Genetic changes can cause increased levels of tolerance, either directly or by affecting the expression of other tolerance genes, thereby allowing bacterial populations to better cope with antimicrobial challenge (1, 3). However, how this tolerance develops and the role of antimicrobials in the evolution of tolerance are not well understood. In the past decade, interest in AMT has increased, particularly upon the discovery of its role in the development of antimicrobial resistance (4). To date, most studies have focused on how AMT develops in Gram-negative model organisms, such as *Escherichia coli* and *Pseudomonas aeruginosa* (4–6). Available studies in Gram-positive bacteria are often retrospective clinical cases where treatment had failed, with the mechanism of tolerance not identified or characterized (7–9).

Natural commensals of the gastrointestinal tract, enterococci are opportunistic pathogens and a leading cause of hospital-acquired infections. Clinical infections are dominated by two species, *Enterococcus faecalis* and *Enterococcus faecium*. The acclimation of enterococci to stressful living environments has significantly contributed to their rise in dominance following the introduction of antimicrobials (10, 11). Enterococci display intrinsic tolerance to a wide variety of clinically relevant antimicrobials (2). The two-component system CroRS and manganese superoxide dismutase SodA have previously been associated with tolerance to cell wall-targeting antimicrobials in *E. faecalis*, in addition to induction of the starvation or stringent response (12–21). However, little is known about how drug-induced AMT may develop.

Teixobactin (TXB) is a new class of antimicrobial with efficacy against multidrug-resistant pathogens, such as vancomycin-resistant enterococci and *Staphylococcus aureus* (22). TXB is a unique depsipeptide that predominantly targets the pyrophosphate-saccharide moiety of lipid II and lipid III, essential precursors of the major cell envelope components peptidoglycan and teichoic acids (22–24). With no reported resistance and the absence of a known naturally occurring resistance cassette, TXB is a promising new antimicrobial for the treatment of multidrug-resistant organisms. However, we have previously shown that *E. faecalis* displays intrinsic tolerance to TXB, while *S. aureus* does not (13), presenting an opportunity to understand how tolerance may contribute to the development of resistance.

Here, we used serial passaging to generate TXB-resistant mutants in three different strains of *E. faecalis*. While no resistant clones emerged, six mutants displaying high-level TXB tolerance were identified, providing a unique opportunity to understand how antimicrobial-induced AMT can develop. Whole-genome sequencing revealed the presence of multiple mutations in each TXB-tolerant isolate. Further characterization of two mutants generated from two different parental strains identified three key pathways: isoprenoid and enterococcal polysaccharide antigen synthesis, and cell envelope stress signaling. Additionally, we show that two of these pathways likely play a role in cross-tolerance between TXB and other clinically relevant cell wall-targeting antimicrobials, highlighting the broader risk of tolerance emerging during antibiotic treatment.

## RESULTS

### Serial passaging of three distinct *E. faecalis* strains from different host origins in the presence of TXB leads to the evolution of antimicrobial tolerance

We have previously shown that *E. faecalis* displays intrinsic tolerance to TXB (13). As AMT has been shown to precede the development of resistance, our first aim was to determine whether prolonged exposure to TXB could lead to the emergence of TXB resistance. To do this, we carried out long-term exposure and serial passaging experiments using three phylogenetically distinct sequence types (ST). ST8 for *E. faecalis* JH2-2 (lab strain), ST108 for AR01/DGVS (veterinary animal isolate), and ST6 for V583 (clinical human isolate). This was to ensure results would be representative of the *E. faecalis* species in general (25–27). Initially, each strain was exposed to TXB, rifampicin, and vancomycin in biological triplicate at concentrations of 0, 0.5, 1, 2, 4, and 8× minimum inhibitory concentration (MIC) for 2 weeks. Cultures were monitored for growth every 48 h and plated on antibiotic-free agar when growth was observed. All cultures treated with the positive control rifampicin showed observable growth after 48 h at all concentrations, with resistance generation confirmed by MIC. Two cultures showed observable growth in the negative control vancomycin at 1× MIC; however, subsequent MIC assays showed no change in vancomycin MIC. No cultures had observable growth at any TXB concentrations ≥1× MIC up to 2 weeks post-challenge. Our focus then shifted to sequential passaging of each strain in triplicate parallel lines in the presence of TXB, with rifampicin and vancomycin again acting as controls. In brief, enterococcal cultures (OD_600_ 0.1) were exposed to an initial sub-MIC (0.25× MIC) concentration of antimicrobial and were sub-cultured into increasing concentrations of antimicrobial (24–48 h) until no growth was observed. Rifampicin-resistant mutants were isolated up to 5× MIC, where passaging was subsequently discontinued. Resistance development was confirmed by MIC assays, showing that the chosen approach was suitable for the evolution of resistant clones. Conversely, no growth was observed in cultures containing >1× MIC of vancomycin, consistent with resistance against this antibiotic typically requiring acquisition of resistance genes (28). In the presence of TXB, each strain was successfully passaged up to 3.75× MIC, but no growth could be obtained at higher concentrations. Of note, *E. faecalis* AR01/DGVS most readily adapted to TXB challenge, growing at higher TXB concentrations earlier than JH2-2 and V583. Resulting cultures (at least one for each strain) were plated on fresh agar containing no antibiotic, and single colonies were picked and assessed for TXB susceptibility.

Of those that displayed an increase in MIC, six isolates in total (two from each strain) were selected for further analysis (Table 1). These will be referred to as A4 and A8 (derived from AR01/DGVS), J4 and J9 (derived from JH2-2), and V3 and V7 (derived from V583) going forward. MIC and minimum bactericidal concentration (MBC) assays were used to identify changes in antimicrobial resistance and tolerance, respectively, to a panel of cell envelope-targeting antimicrobials. All mutants displayed a small increase in TXB MIC from 2 to 4 µg/mL, consistent with their ability to grow 3.75× MIC, but not 4× MIC (8 µg/mL). Interestingly, all mutants displayed a marked increase in tolerance to TXB, penicillin G, and daptomycin compared to their respective wild-type (WT) strains (Table 1). In addition, J4 and J9 showed an increase in tolerance (>16-fold) to ampicillin. These findings were unexpected but highlight that evolution of resistance is very limited in *E. faecalis*, but tolerance is readily obtained upon antibiotic exposure.

**TABLE 1:** Antimicrobial susceptibility of the *E. faecalis* wild types and TXB-tolerant mutant derivatives^*a*^

	Teixobactin		Ampicillin		Penicillin G		Daptomycin	
Strain	MIC	MBC	MIC	MBC	MIC	MBC	MIC	MBC
*E. faecalis* AR01/DGVS	2	16–32	0.5	0.5–1	1	2	1	2–4
A4	4	**128**	0.5–1	1	1	**8**	**2–4**	**8**
A8	4	**128**	0.5–1	1	1–2	**>64**	1–2	**8**
*E. faecalis* JH2-2	2	16–32	0.5–1	0.5–1	1–2	2–4	1	2
J4	4	**64**	**2**	**>128**	2	**>64**	**2**	**4**
J9	4	**64**	0.5–1	**>64**	2	**>64**	**2–4**	**4–8**
*E. faecalis* V583	2	8–16	0.25–0.5	1–2	1	1–2	2	2–4
V3	4	**>128**	0.5–1	1	1	**>32**	1	**4–8**
V7	4	**32–64**	0.5	1–2	2	**>32**	2	**8–16**

^*a*^ MIC and MBC (µg/mL) assay results are representative of the range of at least biological triplicates. Increase in antimicrobial tolerance appears in bold type.

**Fig 1:**
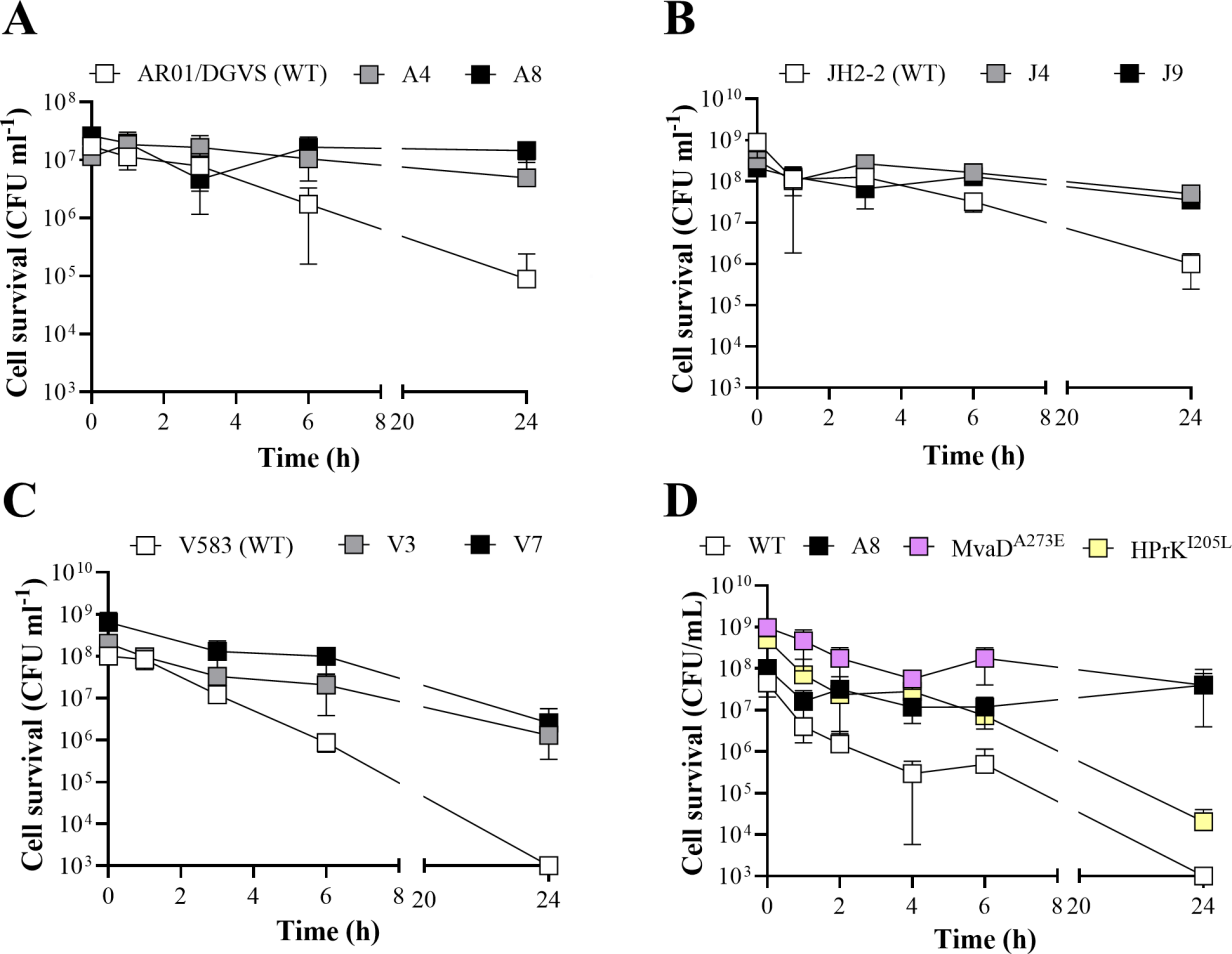
TXB time-kill assay of the *E. faecalis* parent strains and their mutant derivatives. *E. faecalis* WT parent strains and their mutant derivatives (in brackets) (**A**) AR01/DGVS (A4 and A8), (**B**) JH2-2 (J4 and J9), (**C**) V583 (V3 and V7), and (**D**) AR01DGVS WT (A8, MvaD^A263E^, and HPrK^I205L^) were grown to mid-exponential phase (OD_600_ 0.5) in brain-heart infusion (BHI) broth and subsequently challenged with 50 × MIC (100 µg/mL) TXB. Strains were monitored for cell survival as CFU mL^−1^ at time = 0, 1, 3, 6, and 24 h post-challenge. Results are representative of at least biological triplicate and are presented as the mean ± SD.

To determine differences in TXB killing kinetics between the WT and derived TXB-tolerant mutant strains, TXB time-kill assays were carried out at 50× MIC (100 µg/mL) antibiotic concentration. All mutants displayed a reduction in TXB-induced cell killing compared to the WT (Fig. 1). No killing was observed over 24 h in the AR01/DGVS and JH2-2 derived mutant strains (A4, A8, J4, and J9), while a 2-log reduction in cell survival was observed in the respective WT strains after 24 h (Fig. 1A and B). In the V583-derived TXB-tolerant mutants V3 and V7, a 2-log reduction in survival was observed after 24 h, while the viability of the V583 parental strain was reduced to the detection limit (1 × 10^3^ CFU mL^−1^) (Fig. 1C). The difference in the killing kinetics of V583 compared to the other WT strains is consistent with the lower TXB MBC (Table 1). These data show clear differences in killing between the TXB-tolerant mutants and their parental strains, even though two-way analysis of variance (ANOVA) analysis did not report any statistical significance at most time points.

To determine if an extended lag phase may contribute to the observed AMT, growth curves were established for each of the mutants and compared to the parental strain. While all mutants displayed a mild growth defect, except A8 and V7, which grew similarly to their respective parental strains (Fig. S1), it does not mimic the extended lag-phase phenotype previously associated with AMT (4, 29, 30). Taken together, these findings show that serial passaging of *E. faecalis* from diverse genetic backgrounds consistently led to the evolution of high-level TXB tolerance, with some degree of cross-tolerance to other cell envelope-active antimicrobials.

### Whole-genome sequencing of the TXB-tolerant isolates reveals mutations in the cell envelope stress response, isoprenoid and Epa biosynthesis pathways, and carbon metabolism

**Fig 2:**
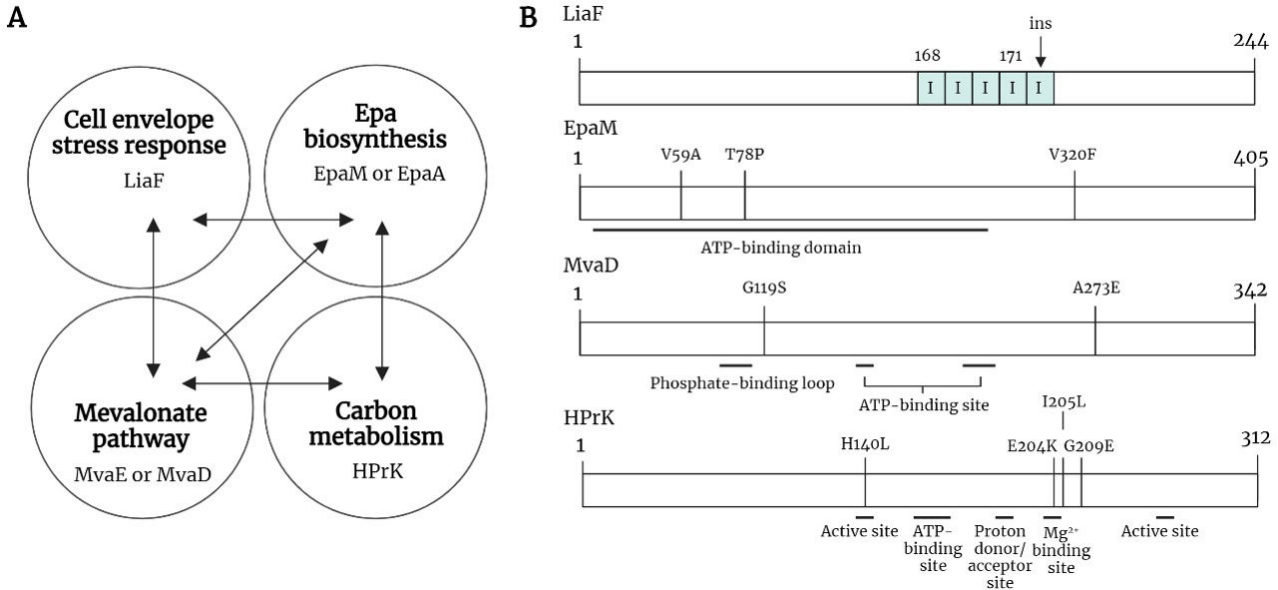
Summary of key genes and pathways with mutations in the TXB-tolerant isolates. (**A**) Mutations were identified in four key pathways: the cell envelope stress response, Epa biosynthesis, carbon metabolism, and the mevalonate pathway. Each isolate contained mutations in at least two of these pathways, with multiple combinations observed. Combinations are represented by arrows between each pathway. (**B**) Multiple mutations were observed in four key genes: *liaF*, *epaM*, *mvaD*, and *hprK*. Mutations were aligned with the protein sequence for each gene, and known or predicted functional sites or domains were annotated using literature searches and UniProt (EpaM, Q832N9; MvaD, Q837E0; HPrK, O07664).

To identify the mutations and subsequent metabolic pathways involved in antimicrobial tolerance evolution, the obtained isolates underwent whole-genome sequencing. Each strain carried multiple mutations, with significant overlap of affected pathways between strains, independent of the origin (Table 2; Fig. 2). Mutations were identified in genes belonging to four key pathways: the cell envelope stress response (*liaF*), the mevalonate pathway (*mvaD, mvaE*), Epa (enterococcal polysaccharide antigen) cell wall polysaccharide biosynthesis (*epaA, epaM*), and carbon metabolism (*hprK*) (Table 2; Fig. 2).

**TABLE 2:** Whole-genome sequencing analysis of the serially passaged *E. faecalis* mutants

Strain^*a*^	Gene affected	V583 locus tag	Annotation	Amino acid change	Mutation type
*E. faecalis* JH2-2					
J4	*epaM*	EF2182	ABC transporter ATP-binding protein	T78P	Missense
	*liaF*	EF2913	Membrane protein	I171ins	Insertion
J9	*epaM*	EF2182	ABC transporter ATP-binding protein	V59A	Missense
	*liaF*	EF2913	Membrane protein	I171ins	Insertion
*E. faecalis* AR01/DGVS					
A4	*mvaE*	EF1364	Acetyl-CoA acetyltransferase/HMG-CoA reductase	G751E	Missense
	*liaF*	EF2913	Membrane protein	I171ins	Insertion
	*mltG*	EF2915	Endolytic transglycosylase	A-T-59 in promoter region	Substitution
A8	*mvaD*	EF0903	Diphosphomevalonate decarboxylase	A273E	Missense
	*hprK*	EF1749	HPr kinase/phosphorylase	I205L	Missense
*E. faecalis* V583					
V3	*mvaD*	EF0903	Diphosphomevalonate decarboxylase	G119S	Missense
	*hprK*	EF1749	HPr kinase/phosphorylase	G209E	Missense
	*epaA*	EF2198	UDP *α*-*N*-acetylglucosaminyl-1-phosphate transferase	Y182D	Missense
V7	*epaM*	EF2182	ABC transporter ATP-binding protein	V320F	Missense
	*hprK*	EF1749	HPr kinase/phosphorylase	H140L; E204K	Missense

^*a*^ Teixobactin-tolerant mutants were from two evolved lineages from three parental strains, *E. faecalis* JH2-2, AR01/DGVS, and V583. Two teixobactin-tolerant mutants were isolated from each parental strain and are designated with a “J,” “A,” or “V,” respectively.

LiaF is a negative regulator of the cell envelope stress response two-component system LiaSR (31–33). We observed an isoleucine insertion at position 171 in LiaF in three of the TXB-tolerant isolates, A4, J4, and J9, increasing the series of isoleucines at this site from four to five (Table 2). Mutations at this site have previously been associated with daptomycin resistance in enterococci but not tolerance at this time (34, 35). This likely explains why we also observed a minor increase in daptomycin resistance in these three isolates (Table 1). In addition to LiaF, amino acid substitutions in EpaM were identified in the J4 (T78P) and J9 (V59A) strains (Table 2). EpaM is the putative ATP-binding protein predicted to form an ABC transporter with EpaL to translocate Epa cell wall polysaccharides across the cell membrane (36–38). A substitution in EpaM was also identified in the *E. faecalis* V583-derived strain V7 alongside two substitutions in HprK (Table 2). HprK is a bifunctional kinase/phosphorylase that phosphorylates and dephosphorylates the conserved regulatory protein HPr in response to carbon availability in *E. faecalis* (39). Variants of HPrK were also identified in V3 and the AR01/DGVS-derived A8 mutant (Table 2). Three mutants, A4, A8, and V3, displayed mutations in genes encoding enzymes of the mevalonate pathway. The mevalonate pathway is responsible for the production of isopentenyl diphosphate (IPP), a key intermediate of isoprenoid and cell envelope biosynthesis (21, 40). Amino acid substitutions were identified in MvaE (A4) and MvaD (A8 and V3), an acetyl-CoA acetyltransferase/HMG-CoA reductase and diphosphomevalonate decarboxylase, respectively. Finally, in mutant A4, in addition to changes to LiaF and MvaE, we also identified a single base-pair substitution in the putative promoter region of *mltG*. MltG is a predicted transmembrane protein with an extracellular enzymatic domain that functions as a lytic transglycosylase and is thought to terminate peptidoglycan biosynthesis (41, 42).

### Substitutions in MvaD but not HPrK drive TXB tolerance

Substitutions in MvaD and HPrK were found to co-occur in two mutants, A8 and V3, from two different parental lineages (AR01/DGVS and V583). To determine whether simultaneous mutations in both genes are required for TXB tolerance, the mutations MvaD^A273E^ and HPrK^I205L^ (identified in A8) were independently re-constructed in the AR01/DGVS parent strain. Growth curves were carried out for each resulting strain, with a slight reduction in growth in the MvaD^A273E^ and a reduction in final cell density in the HPrK^I205L^ strains compared to the A8 mutant and AR01/DGVS wild-type strains (Fig. S2). TXB MIC and MBC assays showed an increase in tolerance in the MvaD^A273E^ but not the HPrK^I205L^ strain (Table 3). In addition, TXB time-kill assays showed a 4-log reduction in survival after 24 h in the WT and HPrK^I205L^ strains, but only a 1-log reduction in the A8 and MvaD^A273E^ strains (Fig. 1D). These data show that the mutations in *mvaD* were likely causative of AMT, while mutations in *hprK* appear to play a minor role, if any. Due to the frequency of mutations identified in this gene in the TXB-tolerant mutants, we cannot rule out that HPrK does contribute to AMT. But with reduced growth, the effect of the *hprK* mutations could be indirect, influencing carbon metabolism or general growth behavior, to support cell survival in conjunction with the tolerance-causative mutations in *mvaD*.

**TABLE 3:** TXB susceptibility of *E. faecalis* strains^*a*^

Strain	TXB		Ampicillin	
	MIC	MBC	MIC	MBC
AR01/DGVS (WT)	2	16–32	n.d	n.d
MvaD^A273E^	**4**	**64**	n.d	n.d
HPrK^I205L^	2	16–32	n.d	n.d
JH2-2 (WT)	2	8	1	1
JH2−2 + pAT28	2	8–16	n.d	n.d
J4	**2–4**	**32–64**	**4**	**>128**
J4 + pAT28	2–4	32–64	2	>128
J4 + pAT28_*epaM*	**2**	**8–16**	**1**	**4**

^*a*^ MIC and MBC (µg/mL) assay results are representative of the range of at least biological triplicate. n.d, not determined. Changes in antimicrobial tolerance appear in bold-type.

### Complementation of the EpaM^T78P^ mutation with EpaM wild-type results in reversion of tolerance to wild-type levels

**Fig 3:**
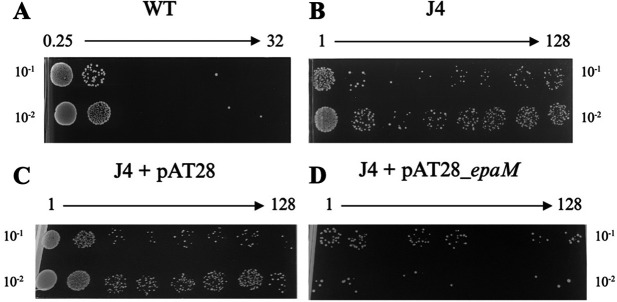
Ampicillin susceptibility assays of the *E. faecalis* JH2-2 wild type (WT), isogenic J4 mutant, and derivatives. Ampicillin MBC assays (μg/mL) were carried out on four *E. faecalis* strains: WT (JH2-2) (**A**), isogenic J4 mutant (**B**), the J4 mutant containing an empty expression vector (J4 + pAT28) (**C**), and the J4 mutant containing an expression vector encoding the *epaM* wild type (**D**). Strains containing the pAT28 vector were supplemented with spectinomycin (500 μg/mL). All results are representative of at least a biological triplicate.

Mutations in genes of the Epa biosynthesis pathway were identified in four of the six TXB-tolerant mutations from two parental lineages (JH2-2 and V583), with all mutations occurring in *epaM* except one. In addition, two of these four mutants also displayed an increase in ampicillin tolerance (J4 and J9). Because of this, the J4 mutant was selected to further investigate the role of EpaM in TXB and ampicillin tolerance. To separate the effects of the two mutations, the J4 mutant (EpaM^T78P^; LiaF^I171^*^ins^*) was complemented with wild-type *epaM* (J4 + pAT28_*epaM*) and tested for TXB and ampicillin susceptibility alongside the J4 mutant, isogenic parental strain (JH2-2), and appropriate vector-only controls (Table 3; Fig. 3). Loss of TXB tolerance and partial loss of ampicillin tolerance was observed in the J4 mutant complemented with a wild-type copy of *epaM* (Table 3; Fig. 3). This was not observed in the J4 mutant + vector only control. This suggests that changes to EpaM, rather than LiaF, are responsible for the increase in TXB tolerance in the J4 mutant, while both *epaM* and *liaF* mutations appear to contribute to ampicillin tolerance. The data also implies that the T78P substitution causes a loss of or reduction in EpaM function, resulting in a decrease in Epa in the cell envelope in this mutant.

**Fig 4:**
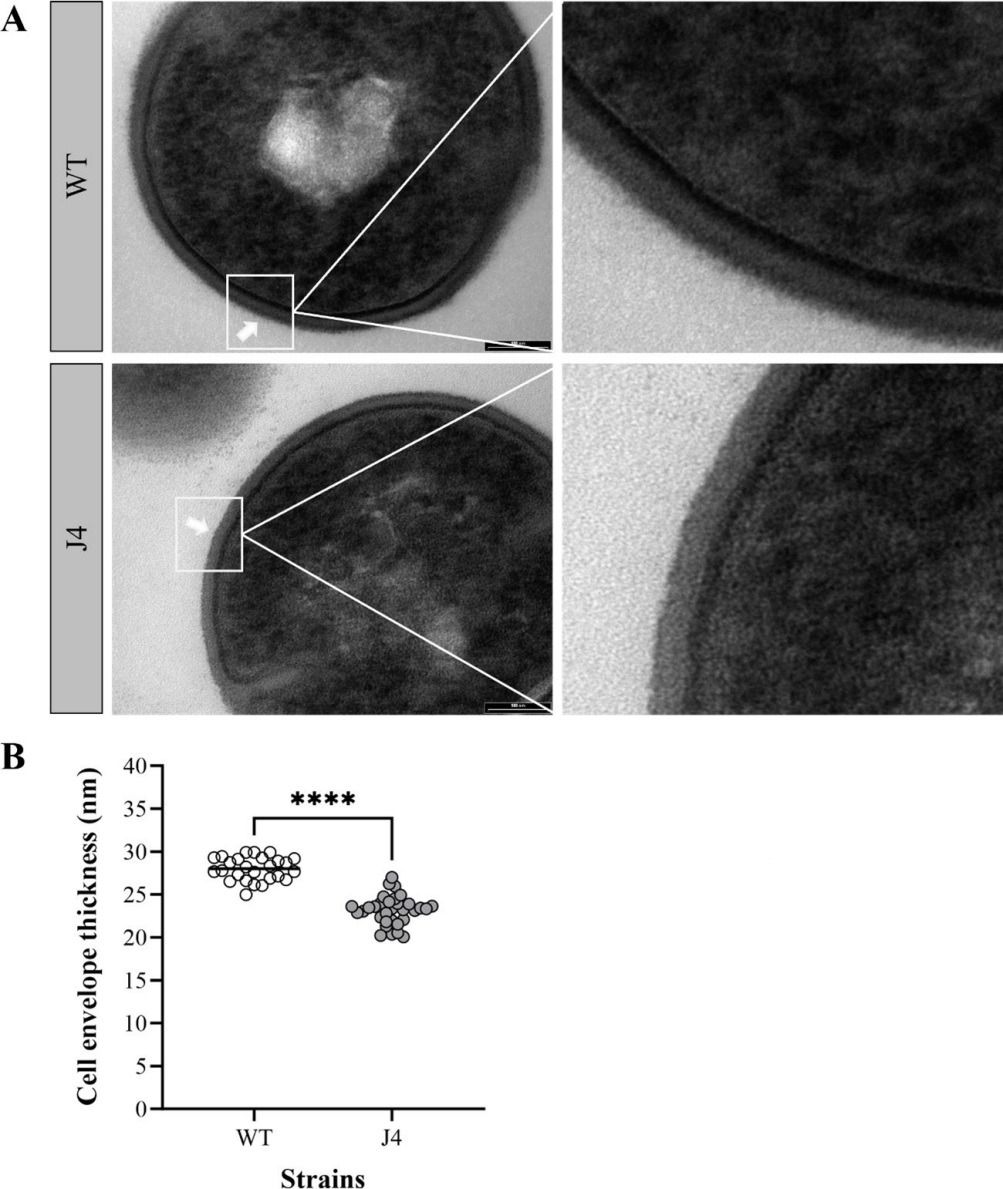
Analysis of the enterococcal cell envelope of the TXB-tolerant J4 mutant and the isogenic parent JH2-2 (WT) using transmission electron microscopy. Strains were grown to mid-exponential phase and imaged at 135 k× magnification by TEM (**A**). Epa was visualized as a separate electron-dense outer layer (white arrows). Following TEM imaging, cells were analyzed for cell envelope thickness (**B**) using ImageJ. Data is presented as the average ± SD. An unpaired *t*-test was used to determine statistical significance; *P* = **** <0.001.

Epa is a major component of the enterococcal cell wall and is visible under the transmission electron microscope (TEM) as a discrete electron-dense outer layer (43). To determine whether mutations in *epaM* result in a decrease in Epa content, we carried out TEM on the *E. faecalis* J4 mutant and parental wild-type strain (JH2-2). First, we observed a decrease in intensity of the dark outer band bordering the thick peptidoglycan layer (Fig. 4A), similar to, albeit less pronounced than, what has previously been observed in a Δ*epaX* Epa-deficient strain (43). Second, analysis of the cell envelope thickness showed a significant reduction in the J4 mutant compared to the parental strain (Fig. 4B). These findings show that the J4-tolerant strain indeed displays alterations in its cell envelope with an overall thinner structure and reduced Epa content.

### The cell envelope stress response is induced in the absence of antimicrobial stress in the TXB-tolerant mutants

**Fig 5:**
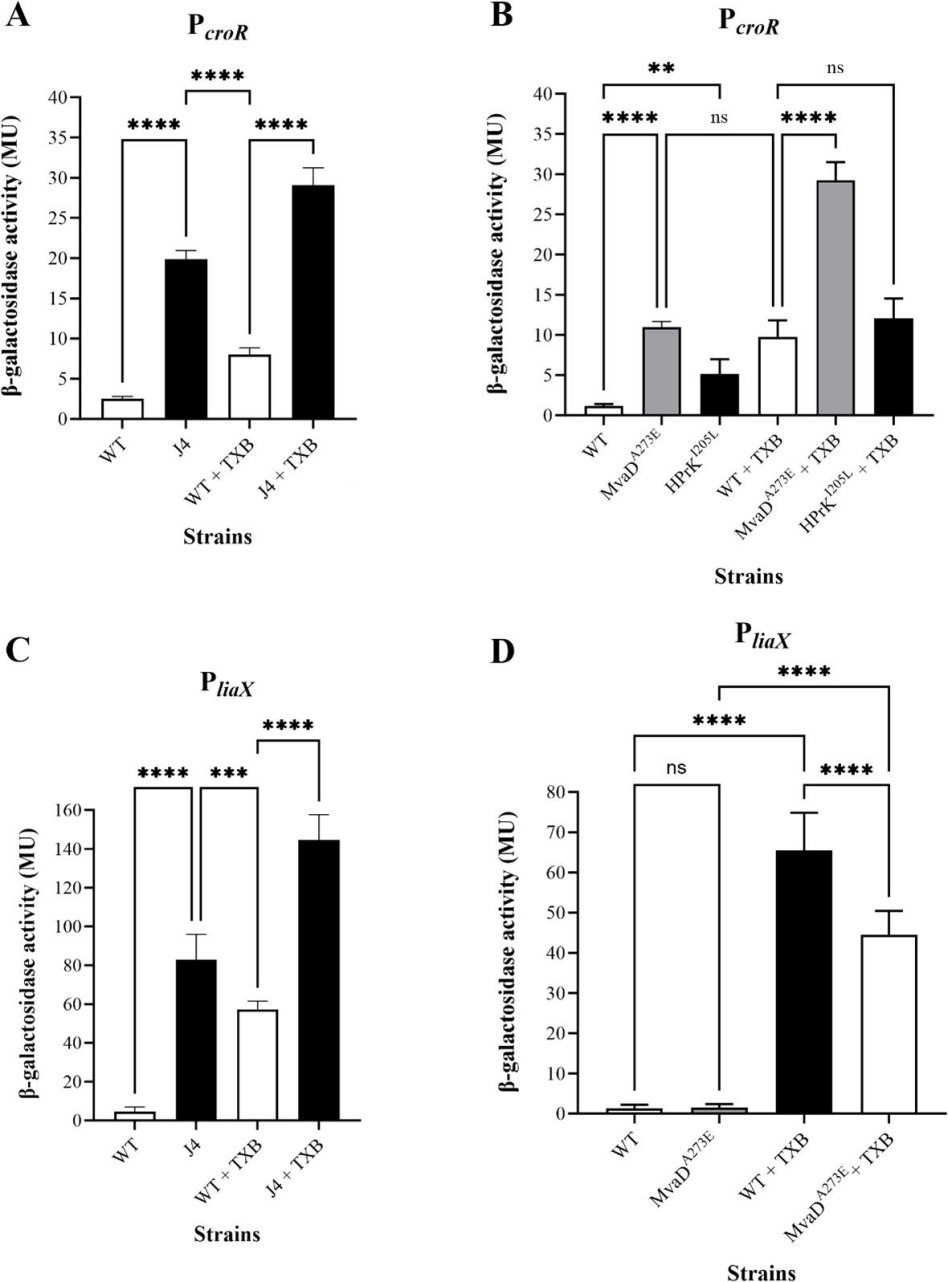
*β*-galactosidase reporter assays of P*_croR_-lacZ* and P*_liaX_-lacZ* expression in the presence and absence of TXB. *E. faecalis* wild-type (WT) strains JH2-2 (**A and C**), AR01/DGVS (**B and D**), and their isogenic mutant strains J4, MvaD^A273E^, and HPrK^I205L^, respectively, were grown to mid-exponential phase (OD_600_ 0.4–0.6). Strains were subsequently challenged with and without TXB (1 µg/mL) for 1 h. Each strain was assayed for *β*-galactosidase activity, representative of CroRS (P*_croR_-lacZ*) (**A and B**) and LiaSR (P*_liaX_-lacZ*) (**C and D**) signaling activity, expressed in Miller units (MU). Data is representative of the mean ± SD of at least biological triplicate. A one-way ANOVA was used to determine statistical significance; *P* = ** <0.01, *** <0.005, **** <0.001; ns, not significant. Some comparisons have been omitted for simplicity.

The mevalonate pathway and EpaM are responsible for producing and transporting core building blocks for the enterococcal cell envelope, consistent with our TEM observations of the J4 mutant. This was a surprising finding, as we expected mutants with increased tolerance to intuitively possess a thicker cell envelope. To understand this phenomenon, we hypothesized that disruptions in the mevalonate and/or Epa biosynthetic pathways may induce the cell envelope stress response. Two key regulators of this response in *E. faecalis* are CroRS and LiaFSR (44). We have previously shown that the cell envelope stress response two-component system CroRS is essential for TXB tolerance in *E. faecalis*, and that both the mevalonate and Epa biosynthesis pathways are upregulated by CroRS in response to TXB treatment (13, 21). With this in mind, we sought to determine whether drug-induced genetic mutations in the mevalonate and Epa pathway induce *croRS* expression. CroRS upregulates expression of its own operon in response to stress; thus, activity of the P*_croR_-lacZ* reporter can be indicative of CroRS signaling activity (21, 45–47). To do this, β-galactosidase reporter assays were carried out by transforming the J4 mutant, the single point variant reconstructions of the A8 strain, HPrK^I205L^ and MvaD^A273E^, as well as their respective wild-type strains JH2-2 and AR01/DGVS with a P*_croR_-lacZ* transcriptional fusion (Table S1). Strains were subsequently grown in the presence or absence of TXB and assayed for CroRS activity. Interestingly, CroRS signaling was activated in all three mutants in the absence of an inducer compared to baseline activity in the uninduced wild-type strains (Fig. 5A and B). CroRS activity in the absence of TXB was ∼10-fold higher in both the J4 and MvaD^A273E^ mutants and fivefold higher in the HPrK^I205L^ mutant compared to their respective wild types (Fig. 5A and B). The addition of TXB led to an increase in P*_croR_-lacZ* activity in all strains tested. However, activity was still approximately threefold higher in both the J4 and MvaD^A273E^ mutant compared to the respective wild-type strains, while activity in the HPrK^I205L^ mutant was similar to its wild type (Fig. 5A and B). This suggests that while mutations in *hprK* may aid in priming the cell for stress by increasing basal CroRS signaling, it is the mutations in *epa* and *mva* that cause an over-activation of CroRS signaling upon challenge with TXB that we propose results in the observed tolerance in these strains.

LiaFSR is an additional cell envelope stress response three-component system upregulated in response to TXB challenge (13). Mutations leading to constitutive activation of this system have previously been associated with daptomycin resistance in enterococci (34, 35). To test if LiaSR signaling activity was affected by the Ile171 insertion in LiaF in the evolved strains, β-galactosidase assays were carried out using a P*_liaX_-lacZ* reporter construct, where *liaX* is the main known target of LiaR (33, 48). This construct was introduced into the J4 mutant and its isogenic wild type and tested in the presence and absence of TXB. The A8 mutant derivative, strain MvaD^A273E^, was used as a non-LiaF^Ile171^*^ins^* encoding TXB-tolerant mutant control, alongside its isogenic wild-type strain.

LiaSR signaling activity was 11-fold higher in the J4 mutant compared to the isogenic wild type in the absence of TXB (Fig. 5C) while there was no significant difference in LiaSR activity between the MvaD^A273E^ mutant and its isogenic wild type (Fig. 5D). This finding was consistent with previous reports linking insertions at the Ile171 position of LiaF with de-repression of LiaSR (34, 49). P*_liaX_-lacZ* activity could be further induced in the J4 mutant upon TXB challenge, resulting in a final twofold higher activity in the J4 strain compared to its isogenic wild type (Fig. 5C). This suggests that the Ile171 insertion does not completely de-repress LiaSR signaling. In the MvaD^A273E^ mutant and its isogenic wild type, addition of TXB led to a significant induction of LiaSR activity, although the final activity was significantly lower in the mutant, compared to its isogenic wild type (Fig. 5D), showing that LiaSR signaling was not de-repressed in this strain. Taken together, these data suggest that while both J4 and the MvaD^A273E^ single variant reconstruction of the A8 mutant showed hyperactive signaling via the CroRS pathway, the J4 mutant additionally contains a partially de-repressed LiaSR signaling system, most likely due to the Ile171 insertion in the LiaF repressor.

### LiaFSR plays a role in TXB tolerance in *E. faecalis*

De-repression and subsequent activation of the LiaSR system in our J4 mutant suggest a putative role in TXB tolerance. To test this, we generated individual Δ*liaR* and Δ*liaS* gene deletions in the *E. faecalis* JH2-2 wild-type parent of the evolved J4 mutant. Subsequent MIC and MBC assays were carried out against TXB, daptomycin, and ampicillin. Deletion of *liaR* and *liaS* resulted in a decrease in daptomycin resistance, as expected (Table 4). Strikingly, while neither deletion affected TXB resistance (i.e., MIC), loss of either gene caused a four-fold reduction in tolerance (i.e., MBC), identifying a novel role for LiaFSR in TXB tolerance (Table 4). Additionally, we observed a loss in daptomycin tolerance with a marked reduction in MBC (Table 4), consistent with previous findings (50). Minor but inconsistent changes in ampicillin susceptibility were observed for both the *liaR* and *liaS* deletion strains, suggesting the LiaFSR system alone was not directly involved in protection against this antibiotic.

**TABLE 4:** Antimicrobial susceptibility testing of the Δ*liaR* and Δ*liaS* deletion strains compared to the isogenic WT (JH2-2)^*a*^

Strain	TXB		Daptomycin		Ampicillin	
	MIC	MBC	MIC	MBC	MIC	MBC
WT	2	16	2	4	0.5	1
∆*liaR*	2	4	1	1	0.25	0.5
∆*liaS*	2	4	1	1	0.5	2

^*a*^ MIC and MBC (μg/mL) assay results are representative of the range of at least biological triplicate. All MICs were carried out in BHI broth due to the lack of consistent growth of the Δ*liaR* strain in Mueller-Hinton broth.

## DISCUSSION

AMT is an important precursor to the development of antimicrobial resistance. However, few studies have investigated how AMT develops, and little is known about the mechanisms of tolerance. *E. faecalis* is a clinically relevant bacterial species that displays intrinsic tolerance to a number of different antimicrobials, including the recently discovered TXB. Our attempt to generate TXB resistance in *E. faecalis* did not result in any resistant isolates but generated six mutants from three different enterococcal backgrounds, which displayed high-level TXB tolerance, as well as varying levels of cross-tolerance to daptomycin and β-lactam antimicrobials. Whole-genome sequencing analysis revealed mutations in four general pathways: the cell envelope stress response (*liaF*), the mevalonate pathway (*mvaD* and *mvaE*), Epa biosynthesis (*epaM* and *epaA*), and carbon metabolism (*hprK*). While we cannot confirm that these are solely drug-induced, and some did not also occur in the “no antibiotic” control, further characterization of these mutants has shown that all pathways except carbon metabolism contribute to an overall increase in TXB tolerance, suggesting they impart a selective advantage under the chosen conditions.

We identified mutations in the mevalonate pathway in half of our high-level TXB-tolerant mutants. We have independently confirmed that mutations in this pathway result in a hyperactive CroRS response and sufficiently confer an increase in TXB tolerance. While reports of mutations in the greater isoprenoid biosynthesis pathway have been associated with altered antimicrobial susceptibility, particularly for proton motive force-dependent antimicrobials, such as gentamicin (51, 52), this is the first direct report (to our knowledge) of a mutation in the mevalonate pathway altering antimicrobial susceptibility in bacteria.

We have previously identified mutations in a heptaprenyl diphosphate synthase (*hppS*), which recover TXB tolerance in a *croRS* deletion mutant (21). HppS uses IPP, a product of the mevalonate pathway and shared precursor of cell wall biosynthesis, as a substrate in demethylmenaquinone (DMK) biosynthesis. We hypothesized that mutations in this gene allow for changes in IPP flux toward cell wall biosynthesis and away from DMK biosynthesis to rescue TXB tolerance (21). It is intriguing that we observe mutations yet again in a pivotal enzyme involved in the isoprenoid biosynthesis pathway correlated with changes in TXB tolerance in *E. faecalis*. While it is currently unclear whether the observed substitutions lead to a gain or loss-of-function in the mevalonate pathway, we do observe an increase in CroRS signaling. Given that CroRS regulates the mevalonate pathway in response to antimicrobial stress (21), it is tempting to speculate that genetic mutations in this pathway in the tolerant strains likely reduce enzymatic activity, triggering a compensatory reaction from CroRS.

Amino acid substitutions in EpaM decreased susceptibility to TXB and ampicillin. Loss-of-function mutations in the Epa pathway have previously been associated with altered susceptibility to cell wall-targeting antimicrobials (2, 53–56). For example, individual gene deletions of *epaI*, *epaOX,* and *epaQ* resulted in an increase in resistance to the β-lactam ceftriaxone in *E. faecalis* (56). Interestingly, the cell envelope stress response two-component system CroRS has previously been shown to significantly contribute to ceftriaxone resistance in *E. faecalis,* while we have highlighted its role in regulating TXB tolerance (13, 21, 46, 57, 58). Here, we observed an upregulation of CroRS in the putative loss-of-function EpaM mutant J4. This suggests, as theorized by Korir et al., that disruptions in the Epa biosynthesis pathway result in cell envelope stress and induction of cell envelope stress response pathways, such as CroRS, leading to a consequential increase in ceftriaxone resistance and TXB tolerance (56).

In our reporter assays, we first observed activation of CroRS signaling in our TXB-tolerant mutants in the absence of antimicrobial stress, and then a further hyper-activation of CroRS signaling upon induction with TXB. From this, we hypothesize a dual mechanism of CroRS activation, one through a malfunctioning CroRS pathway and one in response to cell envelope stress. When induced by both of these factors, CroRS is hyper-activated, increasing expression of its entire regulon, providing a potential common route to AMT in enterococci.

In addition to cell envelope biogenic pathways, we identified an Ile insertion at position 171 in LiaF in 50% of the isolates, a system previously implicated in daptomycin resistance and tolerance (34, 49, 50). A comparison of two tolerant mutants, one encoding the Ile171 insertion (J4) and one encoding the *mvaD* mutation (derived from A8), showed LiaSR hyper-activation in the *liaF* mutant, but not in the *mvaD* mutant. We also show that deletion of either *liaS* or *liaR* results in an increase in susceptibility to killing by TXB. Taken together, our data suggest that Lia activity is not only involved in daptomycin tolerance, but also TXB tolerance.

We observed a striking cross-tolerance between TXB, daptomycin, and ampicillin. While daptomycin tolerance can likely be explained as an effect of Lia dysregulation (34, 49, 59, 60), to our knowledge, this is the first report of evolved ampicillin tolerance in *Enterococcus*. Ampicillin tolerance was only observed in J4 and J9, with both encoding mutations in *epaM* and *liaF*. Mutations in these genes are observed independently in other evolved strains (A4 and V7) but do not result in ampicillin tolerance. In this first instance, this suggests both mutations may be required for ampicillin tolerance. Subsequent complementation of the *epaM* mutation with wild-type *epaM* in the J4 mutant fully restored TXB susceptibility, but only partially fixed ampicillin effects, while individual deletions of *liaR* and *liaS* do not significantly alter ampicillin susceptibility. This suggests both mutations are required to confer ampicillin tolerance.

One final point of discussion is why there is a high frequency of *hprK* mutations but no quantifiable role in AMT. There are a number of potential explanations for this; firstly, we do not know the order of the accumulated mutations in each strain. It is possible that this mutation is required before others can occur, or it has a compensatory function to help accommodate the other mutations needed for tolerance. Secondly, as a key regulator of carbon metabolism in *E. faecalis*, mutations in *hprK* could dysregulate carbon metabolism to its advantage (39). Changes in cell metabolic profiles have been associated with altered susceptibility to antimicrobials in other organisms (61–64). Thirdly, phosphorylated HPr has previously been shown to bind and alter CroR activity in *E. faecalis* (65). Therefore, it is also possible that mutations in *hprK* alter the phosphorylation profile of HPr and thereby its capacity to bind CroR. Such an explanation could be supported by the increase in CroRS-signaling we observe in the HPrK^I205L^ reconstructed mutant in the absence of TXB. Finally, we did not sequence the “no antibiotic” control; therefore, it is possible that some of these mutations may have arisen as an artifact of the serial passaging method in brain-heart infusion (BHI) media rather than the selective pressure of TXB.

Here, we have isolated mutants in the opportunistic pathogen *E. faecalis* that display cross-tolerance to multiple clinically relevant antimicrobials upon selection with a single antibiotic. We propose that mutations in cell envelope biogenesis may have significant direct and indirect consequences on tolerance by decreasing the TXB target and hyperactivating the cell envelope stress response. We hypothesize that indirect hyper-activation of these stress response systems presents a common mechanism of tolerance and suggest it is responsible for the cross-tolerance we observe here. This collective mechanism highlights the importance of understanding mechanisms of AMT and how they develop in order to combat the long-term evolution of antimicrobial resistance. Future work will endeavor to uncover the potential universal role of these cell envelope stress response systems in antimicrobial tolerance across different species.

## MATERIALS AND METHODS

### Bacterial strains

All *E. faecalis* strains were routinely grown in BHI broth and agar at 37°C with no aeration. For selection of the pTCVlac plasmid, kanamycin was added to the media at a concentration of 1,000 μg/mL, while spectinomycin was added to the media at a concentration of 500 μg/mL for selection of the pAT28 plasmid. Teixobactin (Novobiotic) and the synthesized derivative Leu-10 teixobactin (kindly provided by the Velkov Laboratory) were used in this study.

### Spontaneous mutagenesis

#### Long-term exposure

*E. faecalis* AR01/DGVS was grown overnight and diluted to an OD_600_ of 0.05, 0.1, or 0.25 in fresh BHI media. A growth control (no antibiotic) and sterility control (media only) were also included. Cultures were challenged with rifampicin (rif; positive control), vancomycin (van; negative control), and TXB at a range of concentrations: 0.5, 1, 1.25, 1.5, 2, 4, and 8× MIC. Cultures were incubated at 37°C with no agitation for 2 weeks. Growth (if any) was measured every 2 days, and any cultures with growth at concentrations >1× MIC were streaked onto fresh BHI agar and incubated at 37°C overnight prior to MIC and MBC determination.

#### Serial passaging

*E. faecalis* strains AR01/DGVS, JH2-2, and V583 were grown overnight and diluted 1 in 50 (final OD_600_ ∼0.05) into fresh BHI media containing 0.25× MIC (0.5 µg/mL) of TXB and incubated at 37°C with no agitation. *E. faecalis* AR01/DGVS, susceptible to both vancomycin and rifampicin, was also passaged with vancomycin and rifampicin as negative and positive controls, respectively. Cultures were assessed for growth after 24–48 h, and samples with an OD_600_ >0.1 were sub-cultured (1/20 dilution) in fresh BHI media containing antibiotic in incremental steps of 0.5 µg/mL until growth was no longer observed. Cultures displaying growth at >1× MIC of the parental strain were streaked onto fresh BHI agar and incubated at 37°C prior to MIC and MBC determination. Mutants that displayed increased resistance (MIC) or tolerance (MBC) to TXB were sent for whole-genome sequencing (WGS).

### Growth curves

All *E. faecalis* strains were inoculated at a starting OD_600_ of 0.05 in BHI broth and grown at 37°C with no aeration for 8 h. Growth was measured as the optical density at 600 nm every 30 min. Growth curves were carried out using a Varioscan Plate Reader. All bacterial strains used in this study are listed in Table S1.

### Antimicrobial susceptibility assays

MIC and MBC assays were carried out in cation-adjusted Mueller-Hinton broth and BHI agar as previously described, aligning with CLSI guidelines (13). Time-dependent kill assays were carried out to determine killing kinetics over time as previously described (13). Two-way ANOVA using GraphPad Prism Software was used to determine statistical significance.

### Whole-genome sequence analysis

#### Serial passaging

DNA extraction and WGS were performed at the Microbiological Diagnostic Unit Public Health Laboratory, University of Melbourne, Australia. Genomic DNA was extracted from single colonies using a JANUS Chemagic Workstation and Chemagic Viral DNA/RNA kit (PerkinElmer). DNA libraries were created using the Nextera XT DNA preparation kit (Illumina). WGS was performed using the Illumina NextSeq platform with 150 base paired-end reads. Raw read files are available on the European Nucleotide Archive (Project Accession: PRJEB94856).

All WGS analyses were performed using Geneious v8 (http://www.geneious.com). Illumina reads of each genome sequence for each spontaneous mutant were trimmed and paired by name before mapping to reference sequences (*E. faecalis* AR01/DG GenBank: CP022488; *E. faecalis* JH2-2 GenBank: KI518257 and KI518256; *E. faecalis* V583 GenBank: NC_004668). For clear identification of true mutations, sequencing of the parental *E. faecalis* JH2-2 and V583 strains was undertaken simultaneously. Lab reference sequences of *E. faecalis* JH2-2 and V583 were created by mapping to the GenBank JH2-2 and V583 reference sequences prior to analysis of spontaneous mutants. Illumina sequencing reads for each mutant were aligned to their respective parental reference sequence to identify non-synonymous SNPs and indels using Geneious. Genes were annotated with the *E. faecalis* V583 gene homolog (where appropriate) using the NCBI protein BLAST program for continuity. WGS coverage was at least 70× for all strains. To identify SNPs and variations, a threshold of 70% and a minimum of 10 reads was applied. The minimum read coverage per base was 55 with a stdev maximum of ±15.

#### Confirmation of the MvaD^A273E^ and HPrK^I205L^ mutations in *E. faecalis* AR01/DGVS

Isolates were initially screened for the introduction of mutations by PCR amplification and Sanger sequencing. Following the initial confirmation, genomic DNA was extracted from three isolates for each mutant using the DNeasy Blood and Tissue DNA extraction Kit (Qiagen) and sent for whole-genome Illumina sequencing at SeqCenter (Pittsburgh, PA, USA). WGS was performed as written above. WGS analysis was carried out using Geneious Prime 2022.0.2 (https://www.geneious.com) software. Illumina reads were paired using the inbuilt “set-paired reads” function and then trimmed using the “bbduk” plugin in Geneious. Reads were error corrected and normalized using the inbuilt “Error correct and normalize” function to reduce file size and increase mapping efficiency. Reads were mapped to the *E. faecalis* AR01/DG wild-type reference sequence (NZ_CP022488) using the mapper plugin Bowtie2 within the Geneious function “Map to Reference.” Gene mutations were visually confirmed. The inbuilt Geneious function “Find Variations and SNPs” was also used to ensure no additional variations/SNPs had been produced during mutant construction.

### Complementation of EpaM^T78P^ with wild-type EpaM in the teixobactin-tolerant J4 mutant

Wild-type *epaM* was amplified from the parental strain *E. faecalis* JH2-2 genomic DNA and cloned into the complementation vector pAT28 using restriction enzymes *XbaI* and *SphI* to produce pAT28_*epaM* (Table S1). Electrocompetent *E. faecalis* J4 was subsequently transformed with pAT28_*epaM* as previously described. Electrocompetent *E. faecalis* JH2-2 and J4 were also transformed with the empty vector pAT28 as controls.

### Construction of the MvaD^A763E^ and HprK^I205L^ point mutations in the parental wild-type *E. faecalis* AR01/DGVS

Mutant alleles of *mvaD* and *hprK* were amplified from *E. faecalis* A8 genomic DNA and cloned into the pIMAY-Z allelic exchange plasmid by SliCE, as previously described (Table S1) (13, 66, 67). Resulting plasmids pIMAYZ_mvaDA273E and pIMAYZ_hprKI205L were electroporated into *E. faecalis* AR01/DGVS. Mutants were confirmed by WGS.

### Construction of the *croR* and *liaX* promoter-*lacZ* plasmids

The *croR* transcriptional promoter fusion to *lacZ* in *E. faecalis* was constructed in the vector pTCVlac (68). The *croR* promoter sequence was synthesized and cloned into pTCVlac by GenScript via the *EcoRI* and *BamHI* sites in the vector (*croR:*gaattcGATTATTATGATTGGTATAGGTATTTTCGTTCGCTTTTTACCCCCTTTCATCCTTTTTTGGCTATAGTTTATTAACTTTTAGCAAAATCTTCTAAATTTATCGTAATATATCAAGGATTTTGTCCTTTTTAGCAATTATTTGTTAAAATATGGAAAAGAGATTTTTTATATATGGggatcc). The transcriptional promoter fusion P*_liaX_-lacZ* was constructed in the pTCVlac vector as previously described (21).

### β-galactosidase assays

To quantitatively assess induction of the P*_liaX_*- and P*_croR_*-reporter constructs in *E. faecalis*, cells were grown to mid-exponential phase (OD_600_ 0.4–0.5) in BHI medium. Cells were harvested via centrifugation and stored at −20°C. *β*-galactosidase activity was assayed as previously described (21).


$$Miller\;units\;\left(MU\right)=\frac{A420\times 1000}{Time\;\left(minutes\right)\times Volume\;of\;cells\;\left(in\;mL\right)\times OD600}.$$

## SUPPLEMENTAL MATERIAL

Supplemental material aac.00629-25-s0001.docx Table S1; Fig. S1 and S2.

ASM does not own the copyrights to Supplemental Material that may be linked to, or accessed through, an article. The authors have granted ASM a non-exclusive, world-wide license to publish the Supplemental Material files. Please contact the corresponding author directly for reuse.

## References

[1] Windels EM, Van den Bergh B, Michiels J. 2020. Bacteria under antibiotic attack: different strategies for evolutionary adaptation. PLoS Pathog 16:e1008431. doi:10.1371/journal.ppat.1008431 32379814 PMC7205213

[2] Darnell RL, Paxie O, Todd Rose FO, Morris S, Krause AL, Monk IR, Smith MJB, Stinear TP, Cook GM, Gebhard S. 2022. Antimicrobial tolerance and its role in the development of resistance: lessons from enterococci. Adv Microb Physiol 81:25–65. doi:10.1016/bs.ampbs.2022.06.004 36167442

[3] Deventer AT, Stevens CE, Stewart A, Hobbs JK. 2024. Antibiotic tolerance among clinical isolates: mechanisms, detection, prevalence, and significance. Clin Microbiol Rev 37:e0010624. doi:10.1128/cmr.00106-24 39364999 PMC11629620

[4] Levin-Reisman I, Ronin I, Gefen O, Braniss I, Shoresh N, Balaban NQ. 2017. Antibiotic tolerance facilitates the evolution of resistance. Science 355:826–830. doi:10.1126/science.aaj2191 28183996

[5] Fung DKC, Chan EWC, Chin ML, Chan RCY. 2010. Delineation of a bacterial starvation stress response network which can mediate antibiotic tolerance development. Antimicrob Agents Chemother 54:1082–1093. doi:10.1128/AAC.01218-09 20086164 PMC2825962

[6] Liu J, Gefen O, Ronin I, Bar-Meir M, Balaban NQ. 2020. Effect of tolerance on the evolution of antibiotic resistance under drug combinations. Science 367:200–204. doi:10.1126/science.aay3041 31919223

[7] Gu H, Roy S, Zheng X, Gao T, Ma H, Soultan Z, Fortner C, Nangia S, Ren D. 2020. High-level antibiotic tolerance of a clinically isolated Enterococcus faecalis strain. Appl Environ Microbiol 87:1–14. doi:10.1128/AEM.02083-20 PMC7755234 33097497

[8] Kuehl R, Morata L, Meylan S, Mensa J, Soriano A. 2020. When antibiotics fail: a clinical and microbiological perspective on antibiotic tolerance and persistence of Staphylococcus aureus. J Antimicrob Chemother 75:1071–1086. doi:10.1093/jac/dkz559 32016348

[9] Tuomanen E, Durack DT, Tomasz A. 1986. Antibiotic tolerance among clinical isolates of bacteria. Antimicrob Agents Chemother 30:521–527. doi:10.1128/AAC.30.4.521 3539006 PMC176473

[10] Lebreton F, Manson AL, Saavedra JT, Straub TJ, Earl AM, Gilmore MS. 2017. Tracing the enterococci from Paleozoic origins to the hospital. Cell 169:849–861. doi:10.1016/j.cell.2017.04.027 28502769 PMC5499534

[11] Pöntinen AK, Top J, Arredondo-Alonso S, Tonkin-Hill G, Freitas AR, Novais C, Gladstone RA, Pesonen M, Meneses R, Pesonen H, Lees JA, Jamrozy D, Bentley SD, Lanza VF, Torres C, Peixe L, Coque TM, Parkhill J, Schürch AC, Willems RJL, Corander J. 2021. Apparent nosocomial adaptation of Enterococcus faecalis predates the modern hospital era. Nat Commun 12:1523. doi:10.1038/s41467-021-21749-5 33750782 PMC7943827

[12] Bizzini A, Zhao C, Auffray Y, Hartke A. 2009. The Enterococcus faecalis superoxide dismutase is essential for its tolerance to vancomycin and penicillin. J Antimicrob Chemother 64:1196–1202. doi:10.1093/jac/dkp369 19828491

[13] Darnell RL, Knottenbelt MK, Todd Rose FO, Monk IR, Stinear TP, Cook GM. 2019. Genomewide profiling of the Enterococcus faecalis transcriptional response to teixobactin reveals CroRS as an essential regulator of antimicrobial tolerance. mSphere 4:e00228-19. doi:10.1128/mSphere.00228-19 31068434 PMC6506618

[14] Ladjouzi R, Bizzini A, Lebreton F, Sauvageot N, Rincé A, Benachour A, Hartke A. 2013. Analysis of the tolerance of pathogenic enterococci and Staphylococcus aureus to cell wall active antibiotics. J Antimicrob Chemother 68:2083–2091. doi:10.1093/jac/dkt157 23649229

[15] Léger L, Budin-Verneuil A, Cacaci M, Benachour A, Hartke A, Verneuil N. 2019. β-lactam exposure triggers reactive oxygen species formation in Enterococcus faecalis via the respiratory chain component DMK. Cell Rep 29:2184–2191. doi:10.1016/j.celrep.2019.10.080 31747593

[16] Verneuil N, Mazé A, Sanguinetti M, Laplace JM, Benachour A, Auffray Y, Giard JC, Hartke A. 2006. Implication of (Mn)superoxide dismutase of Enterococcus faecalis in oxidative stress responses and survival inside macrophages. Microbiology (Reading) 152:2579–2589. doi:10.1099/mic.0.28922-0 16946253

[17] Abranches J, Martinez AR, Kajfasz JK, Chávez V, Garsin DA, Lemos JA. 2009. The molecular alarmone (p)ppGpp mediates stress responses, vancomycin tolerance, and virulence in Enterococcus faecalis. J Bacteriol 191:2248–2256. doi:10.1128/JB.01726-08 19168608 PMC2655485

[18] Gaca AO, Kajfasz JK, Miller JH, Liu K, Wang JD, Abranches J, Lemos JA. 2013. Basal levels of (p)ppGpp in Enterococcus faecalis: the magic beyond the stringent response. mBio 4:e00646. doi:10.1128/mBio.00646-13 24065631 PMC3781836

[19] Honsa ES, Cooper VS, Mhaissen MN, Frank M, Shaker J, Iverson A, Rubnitz J, Hayden RT, Lee RE, Rock CO, Tuomanen EI, Wolf J, Rosch JW. 2017. RelA mutant Enterococcus faecium with multiantibiotic tolerance arising in an immunocompromised host. mBio 8:e02124-16. doi:10.1128/mBio.02124-16 28049149 PMC5210501

[20] Van Tyne D, Gilmore MS. 2017. Raising the alarmone: within-host evolution of antibiotic-tolerant Enterococcus faecium. mBio 8:e00066-17. doi:10.1128/mBio.00066-17 28223450 PMC5358907

[21] Todd Rose FO, Darnell RL, Morris SM, Rose OE, Paxie O, Campbell G, Cook GM, Gebhard S. 2023. The two-component system CroRS acts as a master regulator of cell envelope homeostasis to confer antimicrobial tolerance in the bacterial pathogen Enterococcus faecalis. Mol Microbiol 120:408–424. doi:10.1111/mmi.15128 37475106 PMC10952268

[22] Ling LL, Schneider T, Peoples AJ, Spoering AL, Engels I, Conlon BP, Mueller A, Schäberle TF, Hughes DE, Epstein S, Jones M, Lazarides L, Steadman VA, Cohen DR, Felix CR, Fetterman KA, Millett WP, Nitti AG, Zullo AM, Chen C, Lewis K. 2015. A new antibiotic kills pathogens without detectable resistance. Nature 517:455–459. doi:10.1038/nature14098 25561178 PMC7414797

[23] Shukla R, Lavore F, Maity S, Derks MGN, Jones CR, Vermeulen BJA, Melcrová A, Morris MA, Becker LM, Wang X, et al.. 2022. Teixobactin kills bacteria by a two-pronged attack on the cell envelope. Nature 608:390–396. doi:10.1038/s41586-022-05019-y 35922513 PMC9365693

[24] Ulm H, Schneider T. 2016. Targeting bactoprenol-coupled cell envelope precursors. Appl Microbiol Biotechnol 100:7815–7825. doi:10.1007/s00253-016-7732-0 27495122

[25] Nallapareddy SR, Duh R-W, Singh KV, Murray BE. 2002. Molecular typing of selected Enterococcus faecalis isolates: pilot study using multilocus sequence typing and pulsed-field gel electrophoresis. J Clin Microbiol 40:868–876. doi:10.1128/JCM.40.3.868-876.2002 11880407 PMC120268

[26] Palmer KL, Godfrey P, Griggs A, Kos VN, Zucker J, Desjardins C, Cerqueira G, Gevers D, Walker S, Wortman J, Feldgarden M, Haas B, Birren B, Gilmore MS. 2012. Comparative genomics of enterococci: variation in Enterococcus faecalis, clade structure in E. faecium, and defining characteristics of E. gallinarum and E. casseliflavus. mBio 3:e00318-11. doi:10.1128/mBio.00318-11 22354958 PMC3374389

[27] Rushton-Green R, Darnell RL, Taiaroa G, Carter GP, Cook GM, Morgan XC. 2019. Agricultural origins of a highly persistent lineage of vancomycin-resistant Enterococcus faecalis in New Zealand. Appl Environ Microbiol 85:e00137-19. doi:10.1128/AEM.00137-19 31028029 PMC6581176

[28] Cetinkaya Y, Falk P, Mayhall CG. 2000. Vancomycin-resistant enterococci. Clin Microbiol Rev 13:686–707. doi:10.1128/CMR.13.4.686 11023964 PMC88957

[29] Fridman O, Goldberg A, Ronin I, Shoresh N, Balaban NQ. 2014. Optimization of lag time underlies antibiotic tolerance in evolved bacterial populations. Nature 513:418–421. doi:10.1038/nature13469 25043002

[30] Sulaiman JE, Lam H. 2021. Evolution of bacterial tolerance under antibiotic treatment and its implications on the development of resistance. Front Microbiol 12:617412. doi:10.3389/fmicb.2021.617412 33717007 PMC7952611

[31] Suntharalingam P, Senadheera MD, Mair RW, Lévesque CM, Cvitkovitch DG. 2009. The LiaFSR system regulates the cell envelope stress response in Streptococcus mutans. J Bacteriol 191:2973–2984. doi:10.1128/JB.01563-08 19251860 PMC2681809

[32] Arias CA, Panesso D, McGrath DM, Qin X, Mojica MF, Miller C, Diaz L, Tran TT, Rincon S, Barbu EM, Reyes J, Roh JH, Lobos E, Sodergren E, Pasqualini R, Arap W, Quinn JP, Shamoo Y, Murray BE, Weinstock GM. 2011. Genetic basis for in vivo daptomycin resistance in enterococci. N Engl J Med 365:892–900. doi:10.1056/NEJMoa1011138 21899450 PMC3205971

[33] Morris SM, Wiens L, Rose O, Fritz G, Rogers T, Gebhard S. 2024. Regulatory interactions between daptomycin- and bacitracin-responsive pathways coordinate the cell envelope antibiotic resistance response of Enterococcus faecalis. Mol Microbiol 121:1148–1163. doi:10.1111/mmi.15264 38646792

[34] Munita JM, Tran TT, Diaz L, Panesso D, Reyes J, Murray BE, Arias CA. 2013. A liaF codon deletion abolishes daptomycin bactericidal activity against vancomycin-resistant Enterococcus faecalis. Antimicrob Agents Chemother 57:2831–2833. doi:10.1128/AAC.00021-13 23507277 PMC3716119

[35] Werth BJ, Steed ME, Ireland CE, Tran TT, Nonejuie P, Murray BE, Rose WE, Sakoulas G, Pogliano J, Arias CA, Rybak MJ. 2014. Defining daptomycin resistance prevention exposures in vancomycin-resistant Enterococcus faecium and E. faecalis. Antimicrob Agents Chemother 58:5253–5261. doi:10.1128/AAC.00098-14 24957825 PMC4135850

[36] Guerardel Y, Sadovskaya I, Maes E, Furlan S, Chapot-Chartier MP, Mesnage S, Rigottier-Gois L, Serror P. 2020. Complete structure of the enterococcal polysaccharide antigen (EPA) of vancomycin-resistant Enterococcus faecalis V583 reveals that EPA decorations are teichoic acids covalently linked to a rhamnopolysaccharide backbone. mBio 11:e00277-20. doi:10.1128/mBio.00277-20 32345640 PMC7188991

[37] Mistou M-Y, Sutcliffe IC, van Sorge NM. 2016. Bacterial glycobiology: rhamnose-containing cell wall polysaccharides in Gram-positive bacteria. FEMS Microbiol Rev 40:464–479. doi:10.1093/femsre/fuw006 26975195 PMC4931226

[38] Teng F, Singh KV, Bourgogne A, Zeng J, Murray BE. 2009. Further characterization of the epa gene cluster and Epa polysaccharides of Enterococcus faecalis. Infect Immun 77:3759–3767. doi:10.1128/IAI.00149-09 19581393 PMC2737988

[39] Kravanja M, Engelmann R, Dossonnet V, Blüggel M, Meyer HE, Frank R, Galinier A, Deutscher J, Schnell N, Hengstenberg W. 1999. The hprK gene of Enterococcus faecalis encodes a novel bifunctional enzyme: the HPr kinase/phosphatase. Mol Microbiol 31:59–66. doi:10.1046/j.1365-2958.1999.01146.x 9987110

[40] Wilding EI, Brown JR, Bryant AP, Chalker AF, Holmes DJ, Ingraham KA, Iordanescu S, So CY, Rosenberg M, Gwynn MN. 2000. Identification, evolution, and essentiality of the mevalonate pathway for isopentenyl diphosphate biosynthesis in gram-positive cocci. J Bacteriol 182:4319–4327. doi:10.1128/JB.182.15.4319-4327.2000 10894743 PMC101949

[41] Iannetta AA, Minton NE, Uitenbroek AA, Little JL, Stanton CR, Kristich CJ, Hicks LM. 2021. IreK-mediated, cell wall-protective phosphorylation in Enterococcus faecalis. J Proteome Res 20:5131–5144. doi:10.1021/acs.jproteome.1c00635 34672600 PMC10037947

[42] Yunck R, Cho H, Bernhardt TG. 2016. Identification of MltG as a potential terminase for peptidoglycan polymerization in bacteria. Mol Microbiol 99:700–718. doi:10.1111/mmi.13258 26507882 PMC4752859

[43] Rigottier-Gois L, Madec C, Navickas A, Matos RC, Akary-Lepage E, Mistou MY, Serror P. 2015. The surface rhamnopolysaccharide epa of Enterococcus faecalis is a key determinant of intestinal colonization. J Infect Dis 211:62–71. doi:10.1093/infdis/jiu402 25035517

[44] Hancock LE, Perego M. 2004. Systematic inactivation and phenotypic characterization of two-component signal transduction systems of Enterococcus faecalis V583. J Bacteriol 186:7951–7958. doi:10.1128/JB.186.23.7951-7958.2004 15547267 PMC529088

[45] Muller C, Le Breton Y, Morin T, Benachour A, Auffray Y, Rincé A. 2006. The response regulator CroR modulates expression of the secreted stress-induced SalB protein in Enterococcus faecalis. J Bacteriol 188:2636–2645. doi:10.1128/JB.188.7.2636-2645.2006 16547051 PMC1428392

[46] Timmler SB, Kellogg SL, Atkinson SN, Little JL, Djorić D, Kristich CJ. 2022. CroR regulates expression of pbp4(5) to promote cephalosporin resistance in Enterococcus faecalis. mBio 13:e0111922. doi:10.1128/mbio.01119-22 35913163 PMC9426447

[47] Kellogg SL, Kristich CJ. 2018. Convergence of PASTA kinase and two-component signaling in response to cell wall stress in Enterococcus faecalis. J Bacteriol 200:e00086-18. doi:10.1128/JB.00086-18 29632091 PMC5971478

[48] Khan A, Davlieva M, Panesso D, Rincon S, Miller WR, Diaz L, Reyes J, Cruz MR, Pemberton O, Nguyen AH, Siegel SD, Planet PJ, Narechania A, Latorre M, Rios R, Singh KV, Ton-That H, Garsin DA, Tran TT, Shamoo Y, Arias CA. 2019. Antimicrobial sensing coupled with cell membrane remodeling mediates antibiotic resistance and virulence in Enterococcus faecalis. Proc Natl Acad Sci USA 116:26925–26932. doi:10.1073/pnas.1916037116 31818937 PMC6936494

[49] Miller C, Kong J, Tran TT, Arias CA, Saxer G, Shamoo Y. 2013. Adaptation of Enterococcus faecalis to daptomycin reveals an ordered progression to resistance. Antimicrob Agents Chemother 57:5373–5383. doi:10.1128/AAC.01473-13 23959318 PMC3811304

[50] Harp JR, Saito HE, Bourdon AK, Reyes J, Arias CA, Campagna SR, Fozo EM. 2016. Exogenous fatty acids protect Enterococcus faecalis from daptomycin-induced membrane stress independently of the response regulator LiaR. Appl Environ Microbiol 82:4410–4420. doi:10.1128/AEM.00933-16 27208105 PMC4959211

[51] Palmer LD, Minor KE, Mettlach JA, Rivera ES, Boyd KL, Caprioli RM, Spraggins JM, Dalebroux ZD, Skaar EP. 2020. Modulating isoprenoid biosynthesis increases lipooligosaccharides and restores Acinetobacter baumannii resistance to host and antibiotic stress. Cell Rep 32:108129. doi:10.1016/j.celrep.2020.108129 32905776 PMC7519801

[52] Sherman MM, Petersen LA, Poulter CD. 1989. Isolation and characterization of isoprene mutants of Escherichia coli. J Bacteriol 171:3619–3628. doi:10.1128/jb.171.7.3619-3628.1989 2661529 PMC210103

[53] Dale JL, Nilson JL, Barnes AMT, Dunny GM. 2017. Restructuring of Enterococcus faecalis biofilm architecture in response to antibiotic-induced stress. NPJ Biofilms Microbiomes 3:15. doi:10.1038/s41522-017-0023-4 28685097 PMC5493694

[54] Furlan S, Matos RC, Kennedy SP, Doublet B, Serror P, Rigottier-Gois L. 2019. Fitness restoration of a genetically tractable Enterococcus faecalis V583 derivative to study decoration-related phenotypes of the enterococcal polysaccharide antigen. mSphere 4:e00310-19. doi:10.1128/mSphere.00310-19 31292230 PMC6620374

[55] Ho K, Huo W, Pas S, Dao R, Palmer KL. 2018. Loss-of-function mutations in epaR confer resistance to ΦnPV1 infection in Enterococcus faecalis OG1RF. Antimicrob Agents Chemother 62:e00758-18. doi:10.1128/AAC.00758-18 30104266 PMC6153818

[56] Korir ML, Dale JL, Dunny GM. 2019. Role of epaQ, a previously uncharacterized Enterococcus faecalis gene, in biofilm development and antimicrobial resistance. J Bacteriol 201:e00078-19. doi:10.1128/JB.00078-19 30910809 PMC6707930

[57] Comenge Y, Quintiliani R, Li L, Dubost L, Brouard J-P, Hugonnet J-E, Arthur M. 2003. The CroRS two-component regulatory system is required for intrinsic β-lactam resistance in Enterococcus faecalis. J Bacteriol 185:7184–7192. doi:10.1128/JB.185.24.7184-7192.2003 14645279 PMC296236

[58] Kellogg SL, Little JL, Hoff JS, Kristich CJ. 2017. Requirement of the CroRS two-component system for resistance to cell wall-targeting antimicrobials in Enterococcus faecium. Antimicrob Agents Chemother 61:e02461-16. doi:10.1128/AAC.02461-16 28223383 PMC5404561

[59] Munita JM, Murray BE, Arias CA. 2014. Daptomycin for the treatment of bacteraemia due to vancomycin-resistant enterococci. Int J Antimicrob Agents 44:387–395. doi:10.1016/j.ijantimicag.2014.08.002 25261158 PMC4417356

[60] Reyes J, Panesso D, Tran TT, Mishra NN, Cruz MR, Munita JM, Singh KV, Yeaman MR, Murray BE, Shamoo Y, Garsin D, Bayer AS, Arias CA. 2015. A liaR deletion restores susceptibility to daptomycin and antimicrobial peptides in multidrug-resistant Enterococcus faecalis. J Infect Dis 211:1317–1325. doi:10.1093/infdis/jiu602 25362197 PMC4402337

[61] Crabbé A, Jensen PØ, Bjarnsholt T, Coenye T. 2019. Antimicrobial tolerance and metabolic adaptations in microbial biofilms. Trends Microbiol 27:850–863. doi:10.1016/j.tim.2019.05.003 31178124

[62] Lopatkin AJ, Bening SC, Manson AL, Stokes JM, Kohanski MA, Badran AH, Earl AM, Cheney NJ, Yang JH, Collins JJ. 2021. Clinically relevant mutations in core metabolic genes confer antibiotic resistance. Science 371:eaba0862. doi:10.1126/science.aba0862 33602825 PMC8285040

[63] Corona F, Martinez JL. 2013. Phenotypic resistance to antibiotics. Antibiotics (Basel) 2:237–255. doi:10.3390/antibiotics2020237 27029301 PMC4790337

[64] Martínez JL, Rojo F. 2011. Metabolic regulation of antibiotic resistance. FEMS Microbiol Rev 35:768–789. doi:10.1111/j.1574-6976.2011.00282.x 21645016

[65] Snyder H, Kellogg SL, Skarda LM, Little JL, Kristich CJ. 2014. Nutritional control of antibiotic resistance via an interface between the phosphotransferase system and a two-component signaling system. Antimicrob Agents Chemother 58:957–965. doi:10.1128/AAC.01919-13 24277024 PMC3910890

[66] Monk IR, Shah IM, Xu M, Tan M-W, Foster TJ. 2012. Transforming the untransformable: application of direct transformation to manipulate genetically Staphylococcus aureus and Staphylococcus epidermidis. mBio 3:e00277-11. doi:10.1128/mBio.00277-11 22434850 PMC3312211

[67] Pidot SJ, Gao W, Buultjens AH, Monk IR, Guerillot R, Carter GP, Lee JYH, Lam MMC, Grayson ML, Ballard SA, Mahony AA, Grabsch EA, Kotsanas D, Korman TM, Coombs GW, Robinson JO, Gonçalves da Silva A, Seemann T, Howden BP, Johnson PDR, Stinear TP. 2018. Increasing tolerance of hospital Enterococcus faecium to handwash alcohols. Sci Transl Med 10:6115. doi:10.1126/scitranslmed.aar6115 30068573

[68] Poyart C, Trieu-Cuot P. 1997. A broad-host-range mobilizable shuttle vector for the construction of transcriptional fusions to β-galactosidase in gram-positive bacteria. FEMS Microbiol Lett 156:193–198. doi:10.1111/j.1574-6968.1997.tb12726.x 9513264

