# Supplementary material for: Prolonged exposure to teixobactin generates cross-tolerance to other cell wall-targeting antimicrobials in Enterococcus faecalis
Source: Antimicrob Agents Chemother. 2025 Nov 18;69(12):e00629-25. doi: 10.1128/aac.00629-25 (PMC12691584; doi:10.1128/aac.00629-25)
Supplement: Supplemental material — Table S1; Fig. S1 and S2. [file aac.00629-25-s0001.docx]

**Table S1. Bacterial strains, plasmids and primers used in this study.**

**
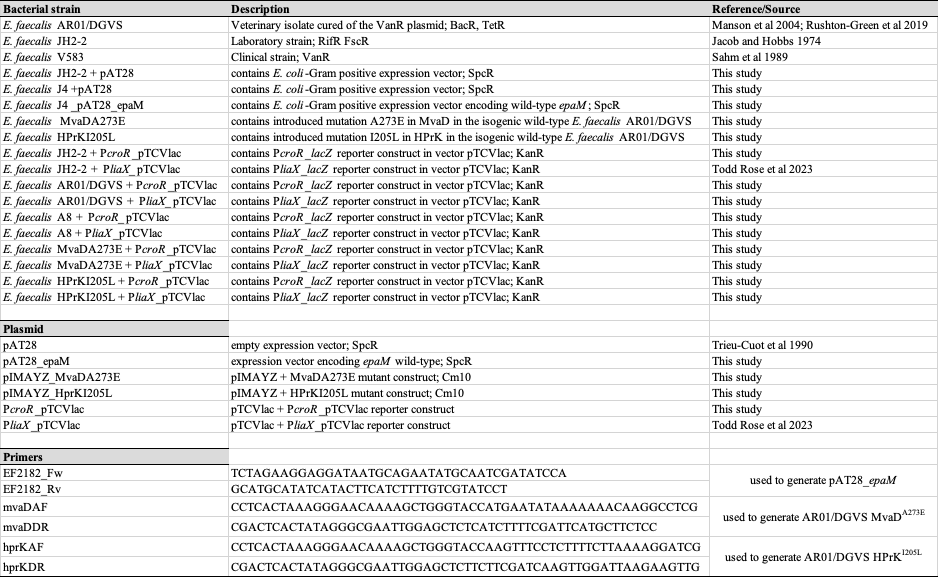
**

**Supplementary Figures**


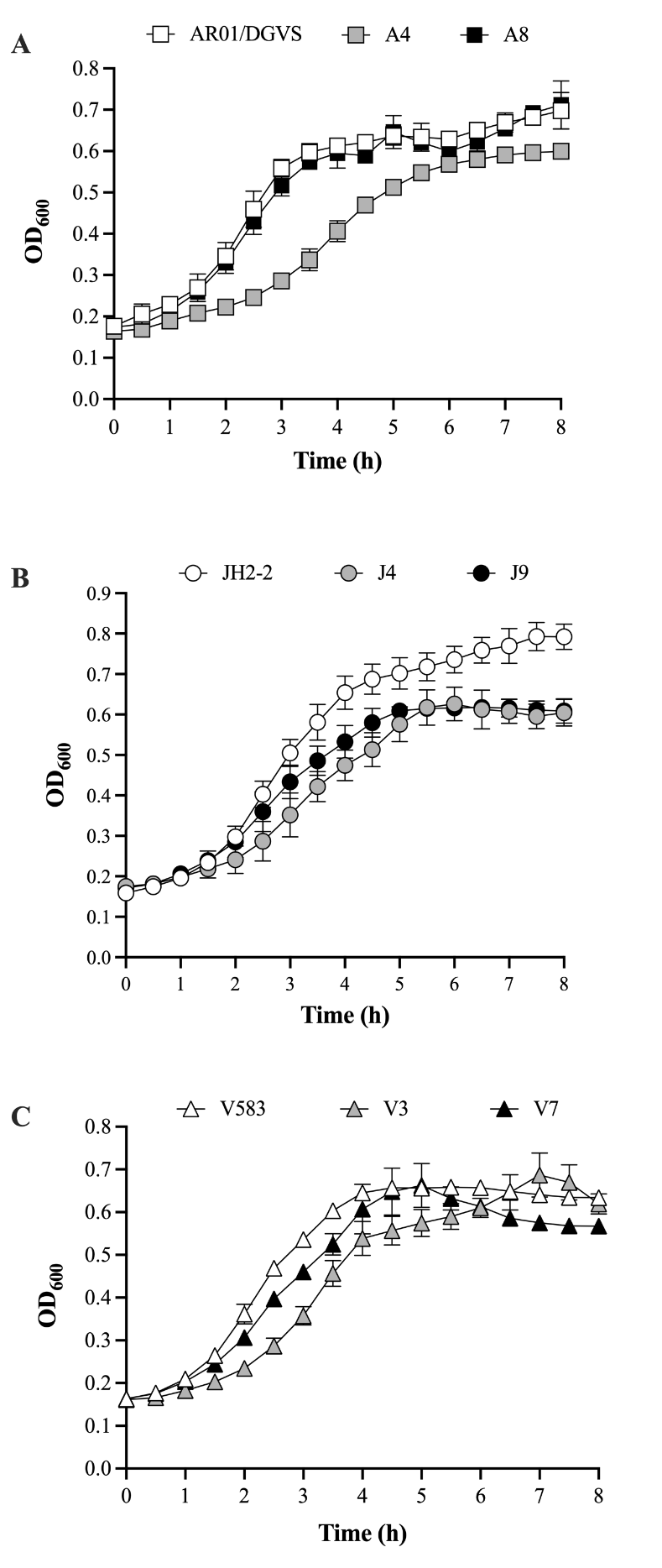


**Figure S1. Growth curves of the *E. faecalis* parent strains (A, AR01/DGVS; B, JH2-2; C, V583) and their mutant derivatives.** Strains were grown in BHI broth at 37$℃$ with no aeration for 8 h. Growth was measured by OD_600_ every 30 mins. TXB-tolerant mutants are plotted alongside their isogenic parent. Data is representative of the mean of biological triplicate ± SD.


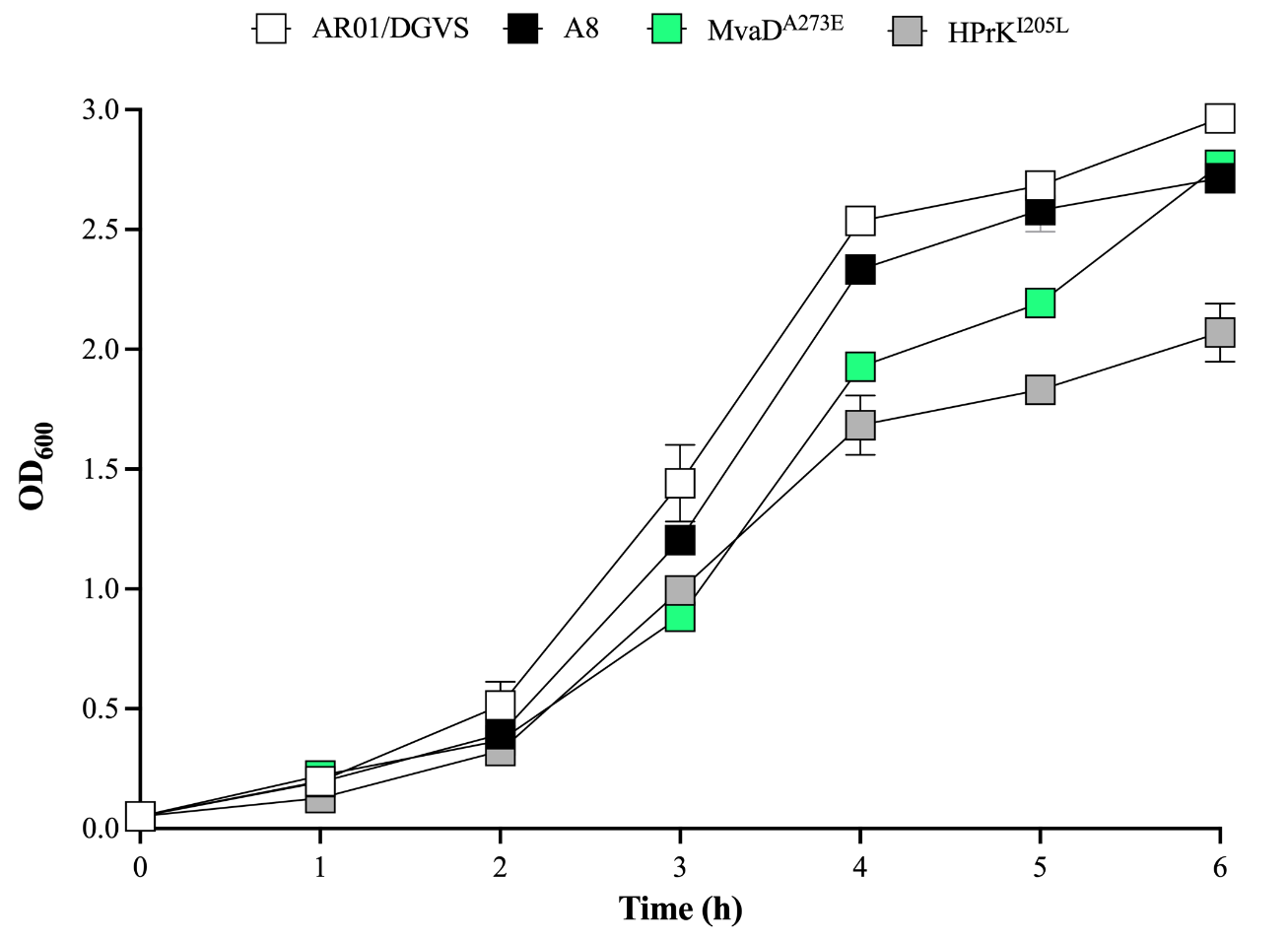


**Figure S2. Growth curve of the *E. faecalis* isogenic WT (AR01/DGVS), the TXB-tolerant A8 mutant and the single point mutation A8-derivatives MvaD^A273^ and HPrK^I205L^.** Strains were grown in BHI broth at 37$℃$ with no aeration for 8 h. Growth was measured by optical density (600 nm) every 1 h until stationary phase was reached at 6 h. Data is representative of the mean of biological triplicate $\pm$ SD.
